# Increased pre-therapeutic serum vascular endothelial growth factor in patients with early clinical relapse of osteosarcoma

**DOI:** 10.1038/sj.bjc.6600201

**Published:** 2002-03-18

**Authors:** M Kaya, T Wada, S Kawaguchi, S Nagoya, T Yamashita, Y Abe, H Hiraga, K Isu, M Shindoh, F Higashino, F Okada, M Tada, S Yamawaki, S Ishii

**Affiliations:** Divison of Orthopedic Surgery and Department of Clinical Research, National Sapporo Hospital, Kikusui 4-2, Shiroishi-ku, Sapporo, 003-0804, Hokkaido, Japan; Department of Orthopedic Surgery, Sapporo Medical University School of Medicine, S-1, W-16, Chuo-ku, Sapporo, 060-8543, Hokkaido, Japan; Department of Oral Pathobiology, Hokkaido University Graduate School of Dental Medicine, N-13, W-7, Kita-ku, Sapporo 060-8586, Hokkaido, Japan; Division of Cancer Pathobiology, Research Section of Pathophysiology, Institute for Genetic Medicine, Hokkaido University, N-15, W-7, Kita-ku, Sapporo, 060-0815, Hokkaido, Japan; Division of Cancer-Related Genes, Research Section of Molecular Pathogenesis, Institute for Genetic Medicine, Hokkaido University, N-15, W-7, Kita-ku, Sapporo, 060-0815, Hokkaido, Japan

**Keywords:** osteosarcoma, pulmonary metastasis, angiogenesis, VEGF

## Abstract

To investigate the clinical significance of circulating angiogenic factors, especially in association with early relapse of osteosarcoma, we quantified pre-therapeutic levels of vascular endothelial growth factor, basic fibroblast growth factor and placenta growth factor in the sera of 16 patients with osteosarcoma using an enzyme-linked immunosorbent assay. After a 1-year follow-up, the serum level of angiogenic factors was analysed with respect to microvessel density of the biopsy specimen and clinical disease relapse. The serum vascular endothelial growth factor levels were positively correlated with the microvessel density with statistical significance (*P*=0.004; Spearman rank correlation) and also significantly higher in seven patients who developed pulmonary metastasis than the remaining nine patients without detectable disease relapse (*P*=0.0009; The Mann–Whitney *U*-test). In contrast, the serum levels of basic fibroblast growth factor or placenta growth factor failed to show significant correlation with the microvessel density or relapse of the disease. Although there was no significant correlation between serum vascular endothelial growth factor levels and the tumour volume, the serum vascular endothelial growth factor levels were significantly higher in patients with a vascular endothelial growth factor-positive tumour than those with a vascular endothelial growth factor-negative tumour. These findings suggest that the pre-therapeutic serum vascular endothelial growth factor level reflects the angiogenic property of primary tumour and may have a predictive value on early disease relapse of osteosarcoma.

*British Journal of Cancer* (2002) **86**, 864–869. DOI: 10.1038/sj/bjc/6600201 www.bjcancer.com

© 2002 Cancer Research UK

## 

Angiogenesis is an absolute requirement for the neoplastic growth of solid tumours after they reach a critical size of 1–2 mm^3^ (Folkman, 1995). It is also essential for tumour metastasis, facilitating the shedding of tumour cells into surrounding blood vessels (Folkman, 1971, 1972, 1990). Tumour cells have been shown to secrete a variety of angiogenic factors, including vascular endothelial growth factor (VEGF), basic fibroblast growth factor (bFGF) and placenta growth factor (PlGF) and thereby induce the local formation of new blood capillaries (Kandel *et al*, 1991; Ferrara *et al*, 1992; Dvorak *et al*, 1995). An association between poor prognosis and increases in vascularity has been reported in a number of tumours, including breast carcinoma (Weidner *et al*, 1991), lung carcinoma (Weidner *et al*, 1993), prostate carcinoma (Macchiarini *et al*, 1992), cervical carcinoma (Smith-McCune and Weidner, 1994) and colon carcinoma (Takahashi *et al*, 1995).

Osteosarcoma is one of the most common malignant bone tumours. Despite recent advances in multimodality treatments consisting of chemotherapy and wide tumour resection, pulmonary metastasis occurs in approximately 50% of the patients with osteosarcoma and remains a major cause of fatal outcome (Rosen *et al*, 1974). Notably, such relapses with pulmonary metastasis or deaths most likely occur during the first year of treatment (Eilber *et al*, 1987; Pratt *et al*, 1990; Souhami *et al*, 1997). Therefore, it is particularly important for the treatment of osteosarcoma to predict the relapse of the tumour at the early phase and customise the protocols.

We previously demonstrated that VEGF expression in osteosarcoma tumour tissue is correlated with high microvessel density, metastatic spread and poor prognosis (Kaya *et al*, 2000). Recently, VEGF has been measured not only in tissues but also in sera by using an enzyme-linked immunosorbent assay (ELISA), and increased serum levels of VEFG have been reported to be correlated with a high incidence of remote metastasis and a poor prognosis in patients with various types of cancer (Salven *et al*, 1997, 1998; Hyodo *et al*, 1998; Jin-no *et al*, 1998; Kumar *et al*, 1998; Landriscina *et al*, 1998; Hara *et al*, 1999; Oehler and Caffier, 2000). Measurement of serum VEGF levels by ELISA appears to be a more promising procedure for quantification than immunohistochemical staining of tissue VEGF, although VEGF can be secreted by megakaryocytes and platelets other than tumour cells (Banks *et al*, 1998; Webb *et al*, 1998; Salven *et al*, 1999; George *et al*, 2000; Lee *et al*, 2000).

In the present study, we investigated the clinical significance of serum VEGF, bFGF, and PlGF in osteosarcoma in a prospective manner, focusing on the correlation with early disease relapse and local angiogenesis. Serum VEGF levels were corrected by platelet counts and the relationship of serum VEGF with tissue VEGF expression as well as the volume of the primary tumours was also analysed.

## MATERIALS AND METHODS

### Patients and sample processing

This study was approved under the institutional guidelines for the use of human subjects in research. Between April 1998 and March 2000 (patients having presented in March 2001 could not be followed up for 1 year, thus the study duration should end in 2000 at latest), all consecutive patients with putative diagnosis of osteosarcoma gave informed consent to provide blood samples. Peripheral venous blood samples were taken from these patients before biopsy and the serum was stored at –80°C until the assays were performed. Among them, patients whose biopsy specimens exhibited histological features compatible to osteosarcoma were entered into the study. There were 16 patients (four women and 12 men) with the age ranging from 20 to 69 years (average 31.2 years). Nine of the tumours were located in the femur, four in the tibia, two in the humerus and one in the pelvis. All tumours were histologically high grade. None of the patients were associated with Paget's disease. Pretreatment work-up studies including palpation of the regional lymph nodes, plain chest X-rays, computed tomography of the lung and abdomen, and bone scintigraphy revealed the development of pulmonary metastasis in two patients. These 16 patients were enrolled into the treatment protocol consisting of neoadjuvant chemotherapy, wide tumour excision, and adjuvant chemotherapy, which was basically a combination of high-dose methotrexate, doxorubicine, cisplatin and ifosmaide. Wide resection margin was achieved in all patients.

Early relapse was defined as the detectable tumour developing in remote sites within 1 year from the onset of the treatments. The cases in which metastatic disease was evident at the onset of the treatments were also defined as early relapse. During a 1-year period, patients were taken plain chest X-rays every month and computed tomography of the lung every 3 months. Bone scintigraphy was taken at 1 year. Consequently, seven out of 16 patients showed early relapse of the disease, all of them being pulmonary metastasis. These patients were divided into two groups, the relapse (seven patients) and no-relapse group (nine patients).

### Immunohistochemical staining

The expression of VEGF and CD34 in the biopsy specimens was determined using the avidin-biotin complex method as described previously (Kaya *et al*, 2000). The primary antibody for VEGF was a rabbit polyclonal antibody (Santa Cruz Biotechnology, Santa Cruz, CA, USA) at 1 : 200 dilution, and the antibody for CD34 was a mouse monoclonal antibody (Nichirei, Tokyo, Japan) at 1 : 100 dilution. The polyclonal antibody reactivity for VEGF with individual tissue sections was considered positive if equivalent staining was seen either in the membrane or the cytoplasm of more than 30% of the tumour cells. The number of CD34-positive vessels was counted in four randomly selected areas of a 1-mm^2^ field and the average number was referred as the microvessel density.

### Evaluation and statistical analysis

Concentration of VEGF, bFGF and PlGF in the sera of patients taken before biopsy was assessed by a commercially available sandwich ELISA (VEGF, IBL, Fujioka, Japan; bFGF and PlGF, R&amp;D Systems, Minneapolis, MN, USA). The serum levels of each angiogenic factor were analysed with respect to (i) the correlation with microvessel density in the biopsy specimen and (ii) the difference between the relapse group and the no-relapse group. The serum VEGF level corrected for the number of platelet before biopsy was also included in the analysis. In addition, the correlation of the serum level of VEGF with (i) volume of the primary tumour and (ii) expression of VEGF in the biopsy specimens were analysed. The volume of the primary tumour was assessed upon the longitudinal and transverse images of magnetic resonance imaging that had been taken before biopsy, and calculated with the following formula: π/6×height×width×depth.

Spearman rank correlation test was used for the analysis of the correlation between the serum levels of each angiogenic factor and the microvessel density, between the serum VEGF level and the tumour volume, and between the serum VEGF level and patient age. The Mann–Whitney *U*-test was used for the comparative analysis of each angiogenic factor levels between the relapse group and no-relapse group and between the VEGF-positive group and the VEGF-negative group in evaluation of the biopsy specimen. Statistical significance was defined as *P*<0.05.

## RESULTS

### Clinical parameters of 16 patients with osteosarcoma

Table 1

**Table 1:**
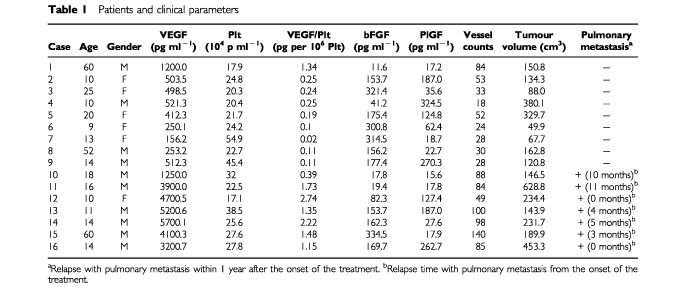
Patients and clinical parameters

summarises the clinical parameters of individual patients. The average serum levels and the 95% confidence interval (CI) of each angiogenic factor were following, VEGF; 1069.4 pg ml^−1^ (95% CI=551.0–2075.5 pg ml^−1^), bFGF; 109.7 pg ml^−1^ (95% CI=61.1–196.8 pg ml^−1^), PlGF; 60.0 pg ml^−1^ (95% CI=32.2–111.9 pg ml^−1^). To determine the distribution of values, means and confidence intervals on the log-transformed data have been back-transformed to give estimates on the untransformed scale.

Two out of 16 patients had pulmonary metastases at the time of the diagnosis and five patients relapsed with pulmonary metastasis within 1 year after the onset of the treatments.

### Serum angiogenic factors concentration and microvessel density

**Figure 1:**
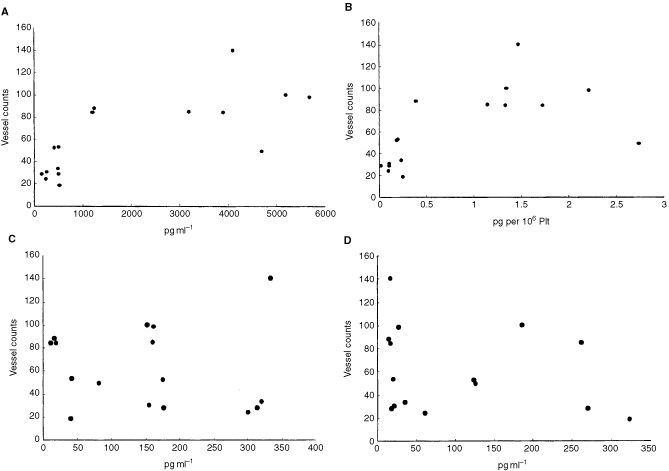
Relationship between microvessel count and serum angiogenic factors levels. Serum angiogenic factors levels against the vessel counts in 16 patients with osteosarcoma. (**A**) VEGF, (**B**) VEGF corrected for platelet counts (**C**) bFGF and (**D**) PlGF. The microvessel counts of the primary osteosarcoma tumours correlated with serum VEGF level (*P*=0.004). and serum VEGF/Plt level (*P*=0.0057). In contrast, no statistically significant correlation was observed between the serum PlGF or serum bFGF level and microvessel density.

We first analysed the correlation between the serum levels of angiogenic factors and the microvessel density in the biopsy specimen of the primary tumour. As shown in Figure 1A

, there was a significant correlation between serum VEGF level and vessel counts within the osteosarcoma tumour (Figure 1A, *P*=0.004; Spearman rank correlation). The correlation remained significant when the serum VEGF levels were corrected by platelet counts (Figure 1B, *P*=0.0057). In contrast, no statistically significant correlation was observed between the serum PlGF or serum bFGF level and microvessel density (Figure 1C, D, PlGF: *P*=0.169; bFGF: *P*=0.529). These results indicate that the serum VEGF level reflects the microvessel density of the primary osteosarcoma tumour. There was no correlation between the serum VEGF level and patient age (*P*=0.898).

### Serum angiogenic factors concentration and early disease relapse

**Figure 2:**
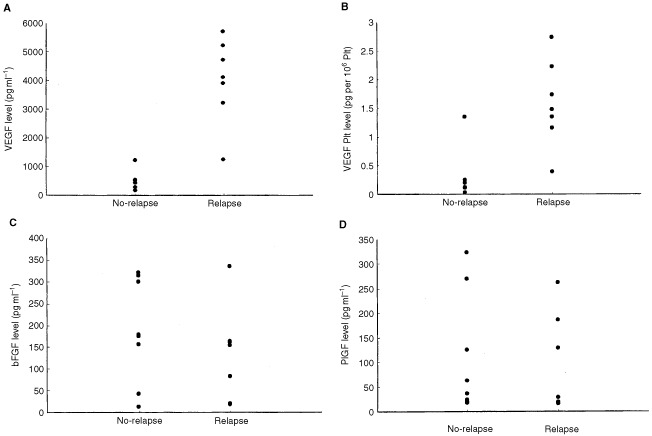
Relationship between the disease relapse and serum concentration of each angiogenic factor. The differences in each angiogenic factor level between the no-relapse and relapse group. The serum levels of VEGF and the serum levels of VEGF corrected for the count of platelet were significantly higher in the patients of the relapse group. In contrast, there was no significant differences in the serum PlGF or serum bFGF level between the two groups.

We next assessed the association between the serum angiogenic factor levels and early relapse of osteosarcoma. The serum VEGF level was significantly higher in the relapse group than that in the no-relapse group (Figure 2A

, *P*=0.0009; Mann–Whitney *U*-test). The above-mentioned mean value of serum VEGF (1069.4 pg ml^−1^) was chosen as the cut-off category, yielding the sensitivity of 100% and the specificity of 88.9% for detecting early disease relapse. The significance was retained after the serum VEGF level was corrected for platelet counts (Figure 2B, *P*=0.0018). In contrast, there was no significant differences in the serum PlGF or serum bFGF level between the two groups (Figure 2C, D, PlGF: *P*=0.458; bFGF: *P*=0.397).

### Serum VEGF levels, tumour volume andtissue VEGF expression

**Figure 3:**
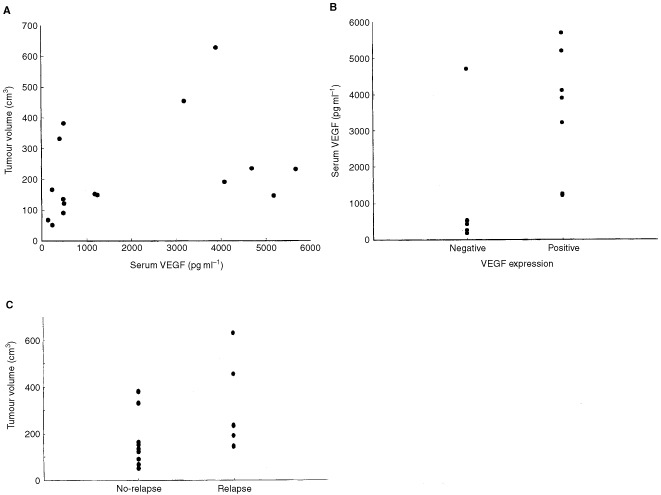
Relationship between the tumour volume or tumour VEGF expression serum VEGF level and disease relapse. (**A**) Relationship between the serum VEGF level and tumour volume. (**B**) Relationship between serum concentration of VEGF and *in vivo* expression of VEGF protein in the primary osteosarcoma tumour. (**C**) Relationship between the disease relapse and tumour volume.

To clarify whether the serum level of VEGF is dependent on the quantitative or qualitative aspects of the primary tumour, we assessed the relationship between the serum VEGF level and the tumour volume as well as the tissue VEGF expression. As shown in Figure 3A

, there was no significant correlation between the serum VEGF level and the tumour volume. In contrast, the serum VEGF levels were significantly higher in patients with a VEGF-positive tumour than those with a VEGF-negative tumour (Figure 3B, *P*=0.005). There was no significant differences in the tumour volume between the relapse and no-relapse groups (Figure 3C, *P*=0.064).

## DISCUSSION

In the current study, we have found that increased pre-therapeutic serum levels of VEGF in 16 patients with osteosarcoma correlate with (i) high microvessel density of the primary tumour, (ii) relapse with pulmonary metastasis during the first year of treatment, and (iii) positive expression of tissue VEGF. In contrast, serum bFGF or PlGF failed to show a positive correlation with the microvessel density or a significant association with relapse of the disease. These findings suggest that the pre-therapeutic serum VEGF levels reflect the angiogenic property of primary tumour and may have a predictive value on early disease relapse of osteosarcoma.

It is known that the majority of patients with osteosarcoma have micrometastatic diseases on initial presentation (Goorin *et al*, 1985; Eliber and Rosen, 1989), leading to early clinical relapse of the tumour in approximately 50% of the patients despite introduction of neoadjuvant chemotherapy (Rosen *et al*, 1974; Eilber *et al*, 1987; Pratt *et al*, 1990; Souhami *et al*, 1997). This fact implies the importance of identifying patients at high risk of relapse early in the course of disease, to whom a more intense chemotherapy or other therapeutic modalities are applied. Accordingly, an attempt has been made to switch the chemotherapy regimen into an alternative one with intensification of reagents before surgery in patients who exhibit progressive disease during neoadjuvant chemotherapy (Wada *et al*, 1996; Meyer *et al*, 2001). It appears to be more ideal to find a biologic profile of osteosarcoma, available at diagnosis, which is linked to relapse of the disease and poor prognosis. Measurement of pre-therapeutic serum VEGF is advantageous for its simple and rapid procedure and provides a prognostic value consistent with immunohistochemical (Kaya *et al*, 2000) or genetical detection (Lee *et al*, 1999) of tissue VEGF in biopsy specimens.

Recently, expression of VEGF has been examined in malignant bone and soft tissue tumours other than osteosarcoma and there have been contradictory results. Whereas Yudoh *et al* (2001) found a significant correlation between tissue VEGF expression and poor prognosis in soft tissue sarcomas, Chao *et al* (2001) and Kuhnen *et al* (2000) failed to find such significance. Kawauchi *et al* (1999) documented no prognostic significance of VEGF expression in synovial sarcoma. In chondrosarcomas, expression of VEGF was associated the histological grade (Ayala *et al*, 2000). Because histological grade is the most important, generally accepted, prognostic factor in soft tissue sarcomas as well as in chondrosarcoma, the predictive value of VEGF may be less important in those tumours than that in conventional osteosarcomas that are exclusively high grade in histology.

Contrary to VEGF, serum bFGF or PlGF measurements failed to show a significant correlation with the microvessel density or association with relapse of the disease, emphasising the critical role of VEGF in angiogenesis of osteosarcoma. Such a predominant role of VEGF than other angiogenic factors has been shown by comparative studies in gastric carcinoma (Yoshikawa *et al*, 2000) and renal cell carcinoma (Edgren *et al*, 1999). On the other hand, bFGF but not VEGF has shown prognostic relevance for head and neck cancer, suggesting that the dependency of tumoral neovascularisation on angiogenic factors may vary between tumour types (Dietz *et al*, 2000).

Since the values of serum VEGF in drawn blood samples can be increased during clot formation (Banks *et al*, 1998; Webb *et al*, 1998; Salven *et al*, 1999; George *et al*, 2000; Lee *et al*, 2000), it is possible that increased serum VEGF levels seen in the present study may not be a true reflection of tumour angiogenic activity. However, correction of the VEGF levels by platelet counts did not impair the significance in correlation with microvessel density or association with early disease relapse. In addition, the increased serum VEGF levels reflected well with tissue expression of VEGF protein within the primary tumour. It should be noted, in this regard, that one patient exhibited high serum VEGF despite negative tissue VEGF expression. In this patient, we have observed significant reduction of serum VEGF after removal of the primary tumour (Kaya M *et al*, unpublished observation), suggesting a problem in tissue staining procedure or the quality of the tissue sections examined.

Because of the rarity of osteosarcoma, there is a difficulty in designing a prospective study with a large number of participants. Although the median value of serum VEGF (1069.4 pg ml^−1^) chosen as the cut-off category yielded the sensitivity of 100% and the specificity of 88.9% for detecting early disease relapse, generalisation of this value requires additional prospective studies with large sample size as well as the sequential analysis of serum VEGF levels in individual patients at various points including the timing of disease relapse. In this sense, the present analysis serves as a pilot study with the strength in its prospective design and the uniformity of participants and treatment protocol.

In conclusion, the present study revealed the clinical significance of pre-therapeutic serum VEGF levels that were significantly higher in patients with osteosarcoma who relapsed during the first year of treatment, and also provided the basis to establish the anti-angiogenic principles for further therapy targeting patients at high risk of angiogenesis-dependent relapse of osteosarcoma.
